# Response to the Specialist Cheesemakers Association on ‘Microbiological quality of raw drinking milk and unpasteurised dairy products: results from England 2013–2019’

**DOI:** 10.1017/S0950268821000820

**Published:** 2021-04-19

**Authors:** J. McLauchlin, H. Aird, E. Forester, F. Jørgensen, C. Willis

**Affiliations:** 1 Public Health England, Food Water and Environmental Microbiology Services, London, UK; 2 Public Health England, Food, Water and Environmental Microbiology Laboratory, York, UK; 3 Public Health England Porton, Food, Water and Environmental Laboratory, Salisbury, UK; 4 Public Health England, Food, Water and Environmental Microbiology, Salisbury, UK

We thank Percival and colleagues for their comments on response to our paper ‘Microbiological quality of raw drinking milk and unpasteurised dairy products: results from England 2013–2019’ published in *Epidemiology and Infection* in May of 2020 [1]. Following an e-mail correspondence with Dr Percival in 2020 which covered similar ground to that in the letter, two of us (CW and JMcL) virtually met with Dr Percival in October 2020 to discuss her concerns. Although this demonstrates our willingness to engage with industry, it is not possible for us to contact all the individual single interest groups on a pre-publication basis, however reputable these organisations may be. We certainly shared the data in this publication with food regulators who, in any event, have closer ties to trade associations than ourselves. Percival and colleagues should note that the subject of the paper is ‘Microbiological quality of raw drinking milk and unpasteurised dairy products' and not just cheese made from raw milk. The paper concludes by stating ‘This group of products is a concern for public health, and there is a need for continued surveillance and implementation of controls during production and throughout the food chain’: we did not single out cheese from other raw milk products or raw drinking milk itself in this sentence. It is worth noting that there is a very limited range of food products for which there is dietary advice for specific risk groups to avoid consumption. Such advice is in place in England for unpasteurised milk and other unpasteurised dairy products [2, 3] and provides evidence of the public health concerns applied to these products.

Within the published paper [1], we have acknowledged sources of bias in the data presented, and separately described microbiological results generated from samples collected during incidents and outbreaks as compared to those from routine monitoring. We also restricted data to those from testing samples of final product at the point of production and to products during sale: results were not considered from samples collected during production including those of food ingredients. We are aware that the microbiota of such products changes during both the production process and during their shelf-life. The results presented here from final products as well as those before the end of shelf-life reflect those closer to consumption by the consumer than samples taken earlier in the production process and may help with public health testing.

We disagree with Percival and colleagues who state that ‘the decision to include data from investigations of outbreaks, where many samples are taken from sites experiencing acute difficulties, is questionable’. This section appears to us to be very much within the scope of this journal, includes data associated with both unpasteurised milk as well as cheese made from unpasteurised milk and we would not be fulfilling our roles as public health microbiologists by suppressing data on the occurrence of infection or to exclude information on interventions for diseases prevention. The comment that a lack of cases of foodborne illness reported to national databases ‘strongly supports the premise that current microbiological criteria established for raw milk cheeses are working as intended to protect public health’ requires more supporting evidence and it is unclear which of the available microbiological criteria Percival and colleagues are referring to here for cheese, as there are several available (see Table 1 and later discussion), as well as criteria for unpasteurised milk for drinking. Recognition and reporting of incidents and outbreaks will be subject to under-reporting; for example, the reporting rate has been estimated to be about 1 case reported for every 5 and 7 cases in the population for salmonellosis and Shiga toxin-producing *Escherichia coli* (STEC) O157, respectively [8]. Reporting rates are likely to be much lower for diseases such as staphylococcal food poisoning which, together with infections from *Salmonella*, *Listeria monocytogenes* and STEC, Donnelly identified as the major foodborne pathogens causing disease associated with unpasteurised cheese consumption [9]. However, the emphasis of this report is on the results of microbiological tests and not the rates of reporting human disease. To state that data ‘should not be published in a peer-reviewed journal’ appears extreme and rather subverts the peer review process. For a single interest group to be the arbiter of what is and what is not published on the basis of unsubstantiated opinion would be contrary to a transparent scientific process and undermine the dissemination of information for public health disease control.

**Table 1.** Guidelines for generic *E. coli* applicable to cheese made from unpasteurised milk

Publication	Product	Lower *E. coli* limit (borderline) (cfu/g)	Upper *E. coli* limit (unsatisfactory) (cfu/g)	Reference
Health Protection Agency, 2009	Ready-to-eat foods (point of sale)	20	100^a^	[4]
The Specialist Cheesemakers Association, 2015	Unpasteurised hard cheese	N/A	100^b^	[5]
	Unpasteurised soft and semi-soft cheese	N/A	10 000^c^	
Scottish Food Enforcement Liaison Committee, 2019	All cheese (end of production)	N/A	100^d^	[6]
IFST, 2020	Unpasteurised soft cheese	<10 (GMP limit at the end of manufacture)	100 (maximum acceptable level by end of shelf-life)	[7]

GMP, Good Manufacturing Practice; IFST, Institute of Food Science and Technology.

a Review cooking and all hygiene procedures including cleaning. Consider taking investigative samples of food and the food preparation environment. Action should be proportional to levels detected. Regulation (EC) No. 2073/2005 (as amended) contains microbiological criteria for some specific food/*E. coli* combinations and the requirements to be complied with by FBOs.

b Regulation (EC) no. 2073/2005 (as amended) has no criteria for *E. coli* in cheese made from raw milk, it is recommended that these cheese types be routinely tested for *E. coli* and an investigation undertaken if a change in trend is detected. The SCA recommended target for *E. coli* applies at the end of ripening in the case of raw milk hard cheese as the level may be higher during manufacture without it being indicative of poor hygiene.

c The targets for *E. coli* in raw milk soft and semi-soft cheeses should apply during ripening. It should be noted that some cheese varieties may not be able to achieve these targets due to intentionally slow acidification. There is no criterion specified for these cheeses in European regulation.

d A target level of <100 cfu/g is considered to be achievable for some cheese types. Where this is exceeded, further evidence should be provided to verify food safety.

There is a legal requirement for food business operators to implement food safety management systems based on HACCP (Hazard Analysis Critical Control Point). Microbiological testing provides important information for verification of HACCP, although testing alone cannot guarantee the safety of food. Microbiological criteria occur in EU law and are legally enforceable. Microbiological criteria also occur in guidelines which have no legal standing. Guidelines are often controversial but provide a framework for standardisation and interpretation of laboratory results for samples collected at different points in the food chain, as well as providing advice on what remedial actions to take. With respect to guidance on acceptable levels of non-toxigenic *E. coli* in raw milk cheese, we agree that there are no EU process hygiene criteria for levels of *E. coli* in cheese produced from raw milk in EU Regulation (EC) 2073/2005: the reasons given for this by Percival and colleagues are unattributed. Guidelines for levels of non-toxigenic *E. coli* in raw milk cheese are available from several groups (see Table 1) including the Specialist Cheesemakers Association (SCA) themselves. The SCA Guidelines for soft and semi-soft cheese appear at odds with those from elsewhere. However, the advice from all the guidelines outlined in Table 1 appears similar and recommends further investigation where adverse results (including unusual trends) are detected. As with all guidelines, food business operators may choose to disregard them if they are able to validate their HACCP system and demonstrate the safety of their food in other ways.

We reported results from testing cheese made from unpasteurised milk and demonstrate an association between detection of elevated levels of *E. coli* and both elevated levels of coagulase-positive staphylococci, as well as the detection of Shiga-toxin genes, but not with the isolation of *L. monocytogenes* [1]. Although conceding that generic *E. coli* as an indicator can be an imprecise tool, we did not enter into speculation as to the mechanisms for these associations. We are glad that there is agreement with Percival and colleagues that there is a ‘subgroup of manufacturers where efforts to improve hygiene should be concentrated’. Despite the public health risks associated with the consumption of unpasteurised milk and products made from unpasteurised milk, it was not our intention to ‘chastise an entire industry’ but to protect public health. There is a common goal to improve the microbiological quality of these products and we would welcome a peer reviewed publication by Percival and colleagues using the considerable quantity of industry data that must be available amongst members of the SCA which may elucidate further the observations and associations we made in 2020 [1].
